# Hints to Specialists

**Published:** 1885-07

**Authors:** J. T. Kent

**Affiliations:** St. Louis


### HINTS TO SPECIALISTS.

Professor J. T. Kent, A. M., M. D., St. Louis.

A quasi-homœopathic gynæcologist once said to some of our students: "If you undertake to cure these diseases (displacements) with your homœopathic remedies you will fail. I have tried remedies and have never found them of any value. I now replace the uterus and adjust a pessary immediately." In such cases what has become of the law? And yet some specialists cry out that the specialties are not sustained. Shall the common average physician sit down and worship such gynæcology, when he, though not pretending great skill, can do better than the specialist, taking his word for it. This is not to underrate him who uses all his means in the right place for the greatest good. There is room for all the specialties, but our specialists must do better than the common practitioner, or they must not complain of being scolded. We expect that the specialist shall not simply and only know the mechanical portion of his department, but that he shall also be expert in the materia medica of his department. It will do for the average doctor to say, "Oh! you materia medica fellows are experts; we are too busy to learn these fine things;" but it will not do for our specialists to be guilty of ignorance in this department. They must know how to cure with remedies, or they must not lay claim to special qualification. When I talk with a specialist I expect to learn of special indications for remedies, and I am generally disappointed. The specialist has the same pathogenesis to work out his cases by that all have, but he generally relies on somebody's hard work, trying to make them fit his cases, and as a rule it does not apply. Every man who claims special excellence in any one department should search the provings for a therapeutics peculiar to his own demands and build for himself. Several years of hard study will reward his labors and he will know none the less of the accumulated experience of others in the application of these same pathogeneses recorded in works on therapeutics. The specialists stand accused of ignorance of the materia medica;—indeed, they are their own accusers when they acknowledge the demand made upon mechanics for the majority of cases treated. Failure to cure by the materia medica should be the exception in all non-surgical diseases, and when other means are resorted to they should be looked upon as but palliative and not curative. There are instances when it is judicious to palliate, but let no man call these means curative. The curse of Homœopathy is the too free use of palliatives, and this is because of the widespread ignorance of the philosophy of Homœopathy and the materia medica. Doctors use palliatives when they do not know what else to do, as the surgeon cuts off the leg when it is the last resort; had he known how to prevent the disease-processes he would have saved the leg. It is a common practice to apply a support to hold in position a displaced uterus and then begin to build up by medicine. Who is wise enough to know what remedy to administer after the symptoms, the only true expression of the disease, have been removed? Yet this is the way some of our specialists go about it, and then complain that "the law is a failure." There might be some reason in first taking the symptoms by which to select a remedy and then applying a pessary; but to the experienced the folly of this will appear, as it is so well known that the symptoms immediately disappear without mechanics. Support is not needed after the right remedy has been taken two days. Again, if a support be used, one has no evidence of good or bad selection.

The cure of these diseases is possible without support with pure medicinal treatment; the demonstrations are too numerous to deny; then let the specialist lay no claim to proficiency who is not able to do better than the average doctor. It matters not how often a woman is examined, only that she is safely, gently, and permanently cured. The question of frequent examinations is one to be laughed at. But the question arises, first of all, do you cure safely, quickly, and permanently? If the physician can make more out of a patient by making frequent observations, and his patient will stand that kind of business, it is well enough, and he must settle the matter with his own conscience if he have such a thing; but he must not so interfere as to delay recovery which should be more or less rapid in most cases. I have the right to take exception, and to criticise, when women go to specialists and pay enormous sums for the treatment of diseases that should be cured with a few doses of a properly selected homœopathic remedy. These things have occurred, and not with our tyros, but those standing in the lead. I can produce the notes if any man dare dispute it, and the worst part of the whole business is that the greatest pretentions are cloaks to the most profound ignorance. These men are generally too wise (?) to be taught by an American author or teacher. They go on with their circumscribed armamentarium for local use, and the thimbleful of *materia medica*, which is all they have, serves the purpose of homœopathic show. If the representatives of the homœopathic school would learn the polychrests so that they could compare them throughout, the demand for mechanics and local slops would decrease. There should be no fashion in medicine; what was good fifty years ago in the hands of the masters should be just as good to-day, and the deviation comes out of departing from the methods of the early physician who had not the labor-saving and brain-saving machines. If the masters could cure such cases with simply great labor, how much better ought we to do! The high degree of perfection will never come to our specialties so long as the specialists are content with the palliatives now in vogue. I am astonished at the amount of palliation that can come from some of these mechanical supports. But I am never astonished at any great skill in the use of remedies in the hands of our specialists, and I still fail to see any good reason for sending a non-surgical case to a specialist to be treated. When they arrange a family circle of their own to include the materia medica and a correct philosophy, then and not until then can they claim patronage that naturally should fall to the specialist. I fail to see any good reason why a homœopathician should advise a patient to consult a professed homœopathic specialist, whose principal means are those developed and used by the allopathist. If there is any reason to suppose a homœopathic physician can use allopathic tools to a better advantage than the allopathist himself, I fail to see it. If allopathic means are better than ours, why uphold the law which is the *sine qua non* of Homœopathy? If a combination of allopathic and homœopathic means goes better, why not associate with congenial spirits, the eclectics?—*Med. Advance.*

